# Editorial Note: Fuzzy ensemble of fined tuned BERT models for domain-specific sentiment analysis of software engineering dataset

**DOI:** 10.1371/journal.pone.0361744

**Published:** 2026-11-02

**Authors:** The PLOS One Editors

The *PLOS One* Editors issue this Editorial Note to inform readers that this article’s [1] Data Availability statement is updated to:

The dataset used in this paper is freely available online. The sources of data are cited in the paper and can also be obtained from authors. We have added the source to download the data in Section 3.2 and Section 5 of the article. Also we uploaded the data at the following link https://github.com/zeeshan0333/Sent-SE. The license type is updated to CC0–1.0 license.

Specific information regarding how the dataset was used in the article can be found in S1 File.

## Supporting information

S1 File Data Availability Information. Specific information regarding how the dataset was used in the article.
(DOCX)

## References

[1] Anwar Z, Afzal H, Altaf N, Kadry S, Kim J. Fuzzy ensemble of fined tuned BERT models for domain-specific sentiment analysis of software engineering dataset. PLoS One. 2024;19(5):e0300279. doi: 10.1371/journal.pone.0300279 38805433 PMC11132486

