# Supplementary material for: Editorial Note: Fuzzy ensemble of fined tuned BERT models for domain-specific sentiment analysis of software engineering dataset
Source: PLoS One. 2026 Nov 2;21(11):e0361744. doi: 10.1371/journal.pone.0361744 (PMC13726972; doi:10.1371/journal.pone.0361744)
Supplement: Supplementary file 1 [file pone.0361744.s001.docx]

The uploaded dataset consists of six CSV files. The purpose of these files with name is given as under:

A. Train.csv: This file is used to train the model. The source of this file is cited at reference [20].
B. Test.csv: This file is used to test the trained model. The source of this file is cited at reference [20].
C. CodeReview-Processed.csv: This is a benchmark dataset file and is used to check the model effectiveness on the already published and publicly available dataset.
D. JavaLib-Processed.csv: This is a benchmark dataset file and is used to check the model effectiveness on the already published and publicly available dataset.
E. StackOverflow-Processed.csv: This is a benchmark dataset file and is used to check the model effectiveness on the already published and publicly available dataset.
F. Jira-Processed.csv: This is a benchmark dataset file and is used to check the model effectiveness on the already published and publicly available dataset.

None of these files directly related to any figure in the article. However, after applying the different trained models on these files results in the form of accuracy, precision, recall, F1 score and AUC were plotted and given in Fig 6, 7, 8 and 9.

Five different model files have been uploaded to the repository. A brief overview of each model file is given below, and we have also updated readme file on Git repository:

A. [BERTSentAnalysis.ipynb](https://github.com/zeeshan0333/Sent-SE/blob/main/BERTSentAnalysis.ipynb): This is standard BERT model that is modified for the domain specific sentiment analysis. This file is used to train and make prediction from BERT standard and BERT Large model on all of above listed dataset.
B. [BertCNN-Sent.ipynb](https://github.com/zeeshan0333/Sent-SE/blob/main/BertCNN-Sent.ipynb): This is BERT and CNN model that is developed for the domain specific sentiment analysis. This file is used to train and make prediction on all of above listed dataset.
C. [SA-Bert-LSTM-Final.ipynb](https://github.com/zeeshan0333/Sent-SE/blob/main/SA-Bert-LSTM-Final.ipynb): This is BERT and LSTM model that is developed for the domain specific sentiment analysis. This file is used to train and make prediction on all of above listed dataset.
D. [SABertGRU.ipynb](https://github.com/zeeshan0333/Sent-SE/blob/main/SABertGRU.ipynb): This is BERT and LSTM model that is developed for the domain specific sentiment analysis. This file is used to train and make prediction on all of above listed dataset.
E. [GPTSA.ipynb](https://github.com/zeeshan0333/Sent-SE/blob/main/GPTSA.ipynb): This is GPT model that is developed for the domain specific sentiment analysis. This file is used to train and make prediction on all of above listed dataset. The main purpose to develop this model is to compare the propose Fuzzy Ensemble model with the GPT model tuned for domain specific sentiment analysis.

Each of these model returns accuracy, precision, recall, F1 score and AUC and prediction results in the form of CSV file. These files are used to perform statistical analysis (ref Fig 4 and 5).
